# Unlocking the Genetic Blueprint of Erucic Acid Content in Mustard (*Brassica napus*): A Meta‐QTL Exploration

**DOI:** 10.1155/tswj/8457904

**Published:** 2026-03-31

**Authors:** Md. Yeamin Hossain, Md. Ridwanul Islam, Mahfuj Ahmed, Shahanaz Parveen, Kazi Md. Kamrul Huda, Mst. Sufara Akhter Banu, Masashi Inafuku, Md. Harun-Ur-Rashid

**Affiliations:** ^1^ Department of Genetics and Plant Breeding, Sher-e-Bangla Agricultural University, Dhaka, Bangladesh, sau.edu.bd; ^2^ Bangladesh Agricultural Research Council (BARC), Dhaka, Bangladesh; ^3^ Faculty of Agriculture, University of the Ryukyus, Nishihara, Okinawa, Japan, u-ryukyu.ac.jp; ^4^ United Graduate School of Agricultural Sciences, Kagoshima University, Kagoshima, Japan, kagoshima-u.ac.jp

**Keywords:** 3KCS, confidence intervals, erucic acid, meta-QTLs, QTLs, rapeseed

## Abstract

*Brassica napus* is a major oilseed crop used for edible oil and industrial applications. Because high‐erucic acid (EA) levels reduce oil quality, identifying robust loci controlling EA is a priority for breeding low‐EA rapeseed cultivars. Here, we performed a meta‐quantitative trait locus (meta‐QTL; MQTL) analysis using 57 published EA QTLs compiled from 12 independent mapping studies. A consensus genetic map spanning 487.24 cM was constructed with 399 markers across two linkage groups (LgA08 and LgC03). Of the 57 QTLs, 37 were successfully projected onto the consensus map and consolidated into 10 MQTL regions. MQTL confidence intervals (CIs) were substantially narrower than those of the projected QTLs (mean CI 3.75 cM vs. 8.56 cM), representing a 2.28‐fold (56.19%) reduction. Candidate gene mining within MQTL intervals identified 67 genes, including eight prioritized candidates associated directly or indirectly with EA metabolism, located in MQTL1.1, MQTL1.2, MQTL1.3, MQTL2.1, and MQTL2.4. Notably, MQTL1.2 and MQTL2.4 contained KCS/*FAE1*‐pathway candidates encoding 3‐ketoacyl‐CoA synthase (KCS), a key enzyme class in very‐long‐chain fatty acid elongation. The MQTLs and prioritized candidate genes identified here provide a valuable foundation for marker‐assisted selection and future functional validation aimed at developing low‐EA rapeseed cultivars with improved oil quality.

## 1. Introduction

*Brassica napus*, also known as rapeseed in general, is considered an important oilseed crop next to palm and soybean oil. It belongs to the secondary allotetraploid species in the Brassicaceae family, derived from hybridization between *B. rapa* (*n* = 10) and *B. oleracea* (*n* = 9), followed by chromosome doubling, forming *B. napus* (*n* = 19) [1]. Rapeseed with high‐erucic acid (EA) content (≥ 45%) is known as industrial rapeseed, whereas the term “canola” refers to an edible oil crop with low‐EA (< 2%) [2]. Rapeseed serves as a key source of both vegetable oil and high‐quality plant‐based protein, making it a valuable edible oil and feed option for humans and farm animals. The seeds, in particular, play a crucial role, as they are the primary providers of both oil and protein. Due to its fame for high‐quality vegetable oil production, rapeseed is considered a competitor of other oil‐yielding crops. Growers value rapeseed not only for its high‐oil content but also for its oil‐extracted meal, which serves as a nutritious animal feed [3]. This meal contains up to 40% high‐quality protein with an amino acid composition that is nearly optimal [4]. The global domestic consumption of rapeseed oil was 33.58 million metric tons in 2023/24 year, which was gradually higher than the previous years [5], with production rates in the EU (30%), China (23%), Canada (14%), and India (12%). Rapeseed is also an important source of vegetable protein, which is used as a protein supplement for farm animals as rapeseed extraction meal (REM) [2].

EA is a monounsaturated omega‐9 fatty acid with a 22‐carbon chain predominantly found in plants of the *Brassica* genus, which includes rapeseed (*B. napus*). Rapeseed oil and seed oil from the mustard family (Cruciferae) differ from other vegetable oils because they contain substantial amounts of EA, a monounsaturated C_22_ fatty acid [6]. Some varieties of oilseed rape, referred to as high‐EA rapeseed (HEAR), produce triacylglycerol (TAG) rich in EA, a compound useful as an industrial feedstock. However, due to health concerns regarding the consumption of EA, canola varieties, also known as low‐EA rapeseed (LEAR), were developed. These varieties have higher levels of oleic acid and reduced amounts of very long‐chain fatty acids (VLCFAs) [7]. Although EA is a naturally occurring compound in these plants and has industrial uses in products like lubricants, plasticizers, and slip agents, its presence in edible oils raises major concerns due to potential toxicity. Genotypes that lack EA are considered to have the highest nutritional value [8], and such low‐EA genotypes are typically found in *B. napus* and *B. rapa* [6, 9]. A specially developed variant of rapeseed, known as “canola” or the “double low” variety, was created in Canada and is distinguished by its low‐EA content (< 2%) [10]. In contrast, HEAR oil is mostly used for industrial purposes, such as producing erucamide for plastic films, cationic surfactants for detergents, and emollients like erucyl alcohol [11]. Additionally, HEAR oil is used in pharmaceuticals, inks, textiles, and even as a dust mask in swine barns to reduce health impacts [12]. Furthermore, a herbicide‐tolerant rapeseed cultivar with extremely high‐EA levels (~66%) was developed in Canada [13]. Seed erucic acid content (SEAC) is an important factor that determines the suitability of rapeseed oil for various applications, including edible LEAR/canola and industrial HEAR uses. Therefore, optimizing this trait is a primary objective in improving *B. napus* to address differing quality and market requirements [14]. As a result, there has been a growing emphasis among researchers on analyzing the molecular and genetic controls of SEAC to facilitate its accurate manipulation via molecular breeding and similar methodologies.

The level of EA in seeds is a quantitative trait that is influenced by multiple genetic loci, where variation is frequently driven by significant additive effects. Studies grounded in classical genetics and quantitative trait loci reveal that two significant genomic regions on the A8 and C3 linkage groups predominantly control the production of EA in seeds [15, 16]. The observed effects are intricately linked to *FAE1*, a gene expressed in seeds that encodes a fatty acid elongase, which plays a crucial role in the biosynthesis of VLCFAs. In HEAR cultivars, there are two functional copies located on the respective chromosomes, exhibiting a significant degree of sequence similarity [14]. Recent functional evidence strongly reinforces the pivotal role of this pathway: A CRISPR/Cas9‐generated double‐knockout mutant (*BnaA08.fae1* and *BnaC03.fae1*) fails to produce EA in its seeds [14]. Furthermore, various other genes that play crucial roles in the biosynthesis and regulation of EA have been documented. Notably, the overexpression of *Bna.GPAT* in *B. napus* has been shown to significantly enhance SEAC [14]. Despite the consistent detection of these significant loci, the reported positions, confidence intervals, and effect sizes of QTL can differ across various mapping populations, environments, and marker systems. This variability complicates the straightforward application of findings into breeding tools and the implementation of consistent marker‐assisted selection (MAS) [15, 16].

To address these inconsistencies, meta‐QTL (MQTL) analysis offers a robust framework for integrating QTL results from various studies and pinpointing stable, consensus loci, while mitigating uncertainty stemming from genetic background, environmental influences, and variations in marker density [17–23]. Compared with association mapping approaches that may generate false positives due to population structure, MQTL analysis emphasizes cross‐study reproducibility and typically yields narrower confidence intervals, improving the reliability of candidate regions for downstream validation and breeding deployments [19]. Despite the clear relevance of EA as a quality‐defining trait in *B. napus*, a comprehensive MQTL synthesis focused on EA has been lacking. In this study, we compiled QTL reports published between 1998 and 2015 and identified 10 MQTLs associated with EA content and further delineated 67 candidate genes within these MQTL intervals, providing a refined genomic foundation for future functional validation and genomics‐assisted improvement of canola and industrial rapeseed.

## 2. Materials and Methods

### 2.1. Compilation of QTLs Database

Using Google Scholar (https://scholar.google.com) and PubMed (https://www.ncbi.nlm.nih.gov/pubmed), an extensive search of the literature was carried out to get information on QTLs linked to EA from published research. Table 1 presents the QTL information obtained from 12 separate studies. The studies included mapping populations ranging in size from 86 to 348 progenies, including different kinds such as recombinant inbred lines (RILs), backcross (BC) populations, and self‐fertilized inbred (SF) lines. All of these populations were subjected to phenotyping at multiple locations. Linkage groups or chromosomes, QTL position, cross names, the number and kind of biparental populations, the additive effect, flanking or closely connected markers, logarithm of odds (LOD), and the phenotypic variation explained (PVE) or *R* ^2^ values were all collected from each study (Tables S1 and S2). If a study did not provide the precise peak location of a QTL, we estimated the probable positions by calculating the midpoint between the two associated markers. Similarly, Guo et al. [35] formulae (mentioned below) were applied to determine 95% of the CI for a QTL in cases when the CI value is not found in the published study.

**Table 1:** Overview of the QTLs study used for analysis of MQTL for the trait erucic acid.

Mapping population	Population type	Population size	Markers	No. of QTLs	Env	Reference
Tapidor × Ningyou7	RIL	202	SSR, SRAP, SNP, STS, IBP, SSAP, RFLP	11	6	[24]
KenC − 8 × N53 − 2	RIL	348	SSR, SRAP, STS, IFLP	2	8	[25]
Y514 × Y517	RIL	150	SSR, RFLP, SSR, ILP	1	1	[26]
Ningyou7 × Tapidor	RIL	202	SSR, Sine‐SSAP	2	5	[27]
*B*.*n* *a* *p* *u* *s* × *B*.*r* *a* *p* *a*_*o* *l* *e* *i* *f* *e* *r* *a*	DH	86	SSR, InDel	2	2	[28]
GH06 × P174	RIL	183	TRAP	4	1	[29]
APl01 × M083	BC	182	RAPD, SSR, SRAP	2	1	[30]
Mansholts_Hamburger_Raps × Samourai	DH	148	RFLP, AFLP	9	2	[31]
Ningyou7 × Tapidor	RIL	188	STS, SSR, RFLP, AFLP, SNP	16	4	[16]
Varuna × Heera	RIL	123	SNP, RFLP, RAPD, AFLP, SSR	2	1	[32]
MF86 × LE76	SF2	94	SSR	5	2	[33]
B002 × Hokkaido	BC	126	RAPD	1	2	[34]

Abbreviations: AFLP, amplified fragment length polymorphism; Env, environment; IBP, inter‐primer binding site; IFLP, intron fragment length polymorphism; ILP, intron length polymorphism; InDel, insertion‐deletion; RAPD, random amplified polymorphic DNA; RFLP, restriction fragment length polymorphism; Sine‐SSAP, short interspersed nuclear element–sequence‐specific amplified polymorphism; SNP, single nucleotide polymorphism; SRAP, sequence‐related amplified polymorphism; SSAP, sequence‐specific amplified polymorphism; SSR, simple sequence repeat; STS, sequence tagged site.

RIL:

CI cM=163N×R²

BC and *F*_2_ lines:

CI cM=530N×R²

Here, *N* stands for the number of the population, and *R* ^2^ represents the percentage of phenotypic variance that the QTL accounts for.

Following the guidelines in the BioMercator v4.2 handbook [21], two essential data files for each research were produced: (i) a file containing genetic mapping data, and (ii) a file with information on QTL. The genetic map file included details on the kind and size of the population, the purpose of mapping, the map units, and the various marker positions along the linkage groups. The QTL information file included information on the chromosomal names, the characteristics or disorders associated with each QTL, the PVE value for every QTL, as well as the precise locations of every QTL within the genome. Research without essential data, including marker locations and PVE or *R* ^2^ values, was not included in the study.

### 2.2. Building the Consensus Map and Projecting QTLs

To improve the statistical efficiency and precision of QTL discovery, a high‐density genetic linkage map is essential [36]. A high‐density linkage map of *B. napus* that is available to the public was utilized as the reference map in accordance with this principle [37]. There are 403 DArT markers and 568 non‐DArT markers in the reference map. These include 381 SSRs, 69 STSs (with candidate genes), 68 SNPs, 34 RFLPs, 14 markers associated with centromeric sequences, and two AFLP markers. It extends 1,987.2 cM throughout the whole length of the A and C genomes’ 19 chromosomes. This dense distribution corresponds to around 0.88 Mbp of the haploid genome, with an average marker spacing of 1.46 cM [37]. This consensus genetic map also included additional markers flanking each QTL related to EA genes, as reported in individual studies, which were also integrated into this genetic map. With BioMercator v4.2, the QTLProj program was used to project the QTLs into this consensus map [38]. QTLProj uses a dynamic approach to choose the best context for projection. In order to create this ideal context, two common markers that surround the QTL on the original map must be found. Additionally, the distance estimations between the original and reference maps must be consistent. The behavior of the method is determined by two parameters: the minimum value of the ratio of flanking marker interval distances and the minimal *p* value obtained from comparing the original and reference maps′ tests for the homogeneity of these distances [22].

### 2.3. MQTL Analysis

Linkage maps and QTL data were created independently for use in BioMercator v4.2 input files for the MQTL analysis. BioMercator v4.2 (a QTL projection tool) ([17]; https://mybiosoftware.com/biomercator-genetic-maps-qtl-integration.html) was then used to project the QTLs onto the reference map. Using the “Veyrieras” two‐step approach in BioMercator v4.2 [22], the MQTL analysis was carried out. The average weight of evidence criterion (AWE), Bayesian information criterion (BIC), AIC Model 3 (AIC3), Akaike Information Criterion (AIC), and corrected AIC (AICc) are the five models′ selection criteria that the method assesses in the first stage. It chooses the best MQTL model with the lowest criterion value and greatest weight after evaluating the three optimal parameter values. Using this chosen model, the detected MQTLs are visualized in the second stage. Subsequently, the program produces a file with comprehensive details about these MQTLs, such as their locations, CI, and the number of QTLs present inside each MQTL.

### 2.4. CGs Identification Within the MQTL Regions and Expression Analysis

The Ensembl Plant database (https://plants.ensembl.org) was used to establish the physical locations of the markers encompassing the MQTL areas in order to locate potential genes inside them. First, the flanking marker nucleotide sequences of a subset of chosen MQTLs were extracted from the Ensembl Plant database, the information resource of *B. napus* multiomics (https://yanglab.hzau.edu.cn/BnIR), or the relevant scientific literature. Following that, BLAST searches were performed using these nucleotide sequences against the *B. napus* reference genome available in the Brassica Database (http://brassicadb.cn). As a result, each MQTL′s matching genetic area could be identified. The GO terms and their definitions were obtained from the BnIR/BnPIR database (https://yanglab.hzau.edu.cn/BnIR) for use in GO enrichment analysis. We conducted the GO enrichment analysis on the candidate genes using the BnIR/BnPIR website (https://yanglab.hzau.edu.cn/BnIR/GO) and (https://yanglab.hzau.edu.cn/BnIR/KEGG). The WeiShengxin website (http://www.bioinformatics.com.cn) was utilized to visualize the KEGG (enhanced horizontal bars with colors) and GO (biological process [BP], cellular component [CC], and molecular function [MF] three‐in‐one bar plot) results. The targeted CGs′ transcriptome data were sourced from the BnIR/BnPIR (https://yanglab.hzau.edu.cn/BnIR) database. The amount of expression is measured in transcripts per million (TPM). Finally, Tbtools‐II retrieved and presented the highlighted CGs′ expression.

## 3. Result

### 3.1. The Distribution of QTLs From Original Studies

A total of 57 QTLs were chosen from 12 studies to find the common genomic regions linked to EA content. Of the 57 QTLs, 49 were derived from RIL populations, three from BC populations, and five from self‐generation intercross (SFi) populations. On LgC03, there were 17 QTLs, but on LgA08, there were 21 with an average of 7.22 cM and 10.21 cM, respectively. CIs for these 57 QTLs ranged from 0.16 to 27.2 cM on LgA08 and from 0.78 to 29.52 cM on LgC03. Figure 1 shows an overview of the number of QTLs in the respective chromosomes along with their LOD scores and phenotypic variance explained value (PBE value).

Figure 1An overview of the 57 QTLs that were associated with erucic acid content in *B. napus* number of QTL per linkage group (a), phenotypic variance explained (b) and LOD score (c).

**Figure:**
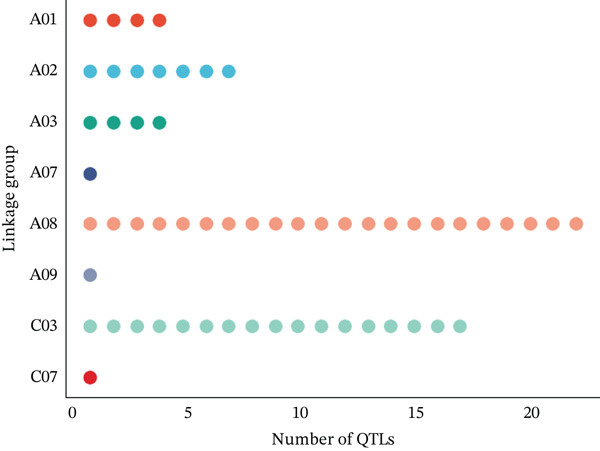
(a)

**Figure:**
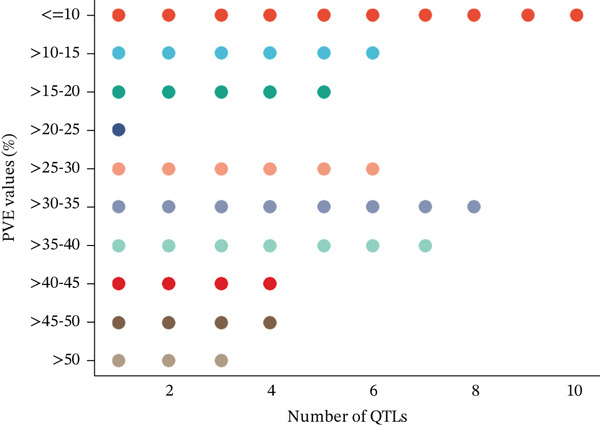
(b)

**Figure:**
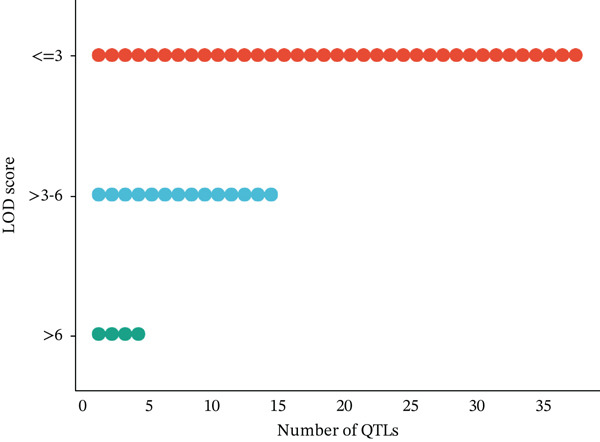
(c)

### 3.2. Projection of QTLs and Consensus Map Construction

The map comprises 399 markers from both linkage groups of *B. napus*, covering a total distance of 487.24 cM in the consensus map. The length of LgA08 was 199.37 cM, and LgC03 was 287.87 cM. The number of markers for LgA08 was 147, and for LgC03 it was 252. Additionally, the average marker density in linkage Group 8 was 0.73 marker/cM, whereas linkage Group 13 had an average of 0.87 marker/cM. The software only used 37 QTLs for projection out of the 12 studies reporting 57 QTLs (Figure 2).

Figure 2QTL distribution on the consensus genetic map projected in the LgA08 (a) and LgC03 (b). Bars on the left side of the chromosome indicate the QTL. Marker density inside a chromosome is shown by black bars. cM represents the genetic distance, which is shown by the numbers on the right side of the chromosome.

**Figure:**
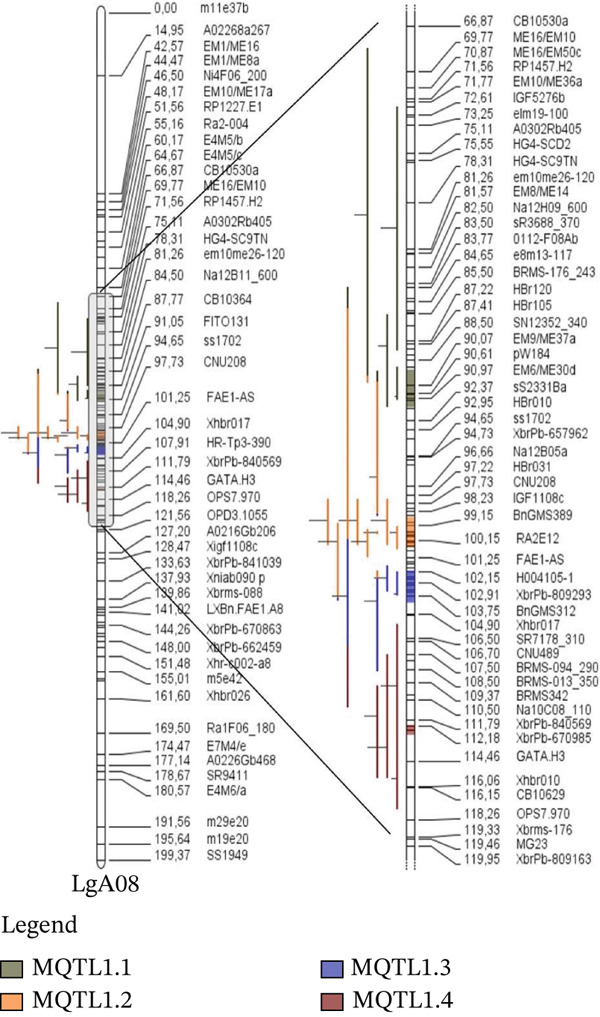
(a)

**Figure:**
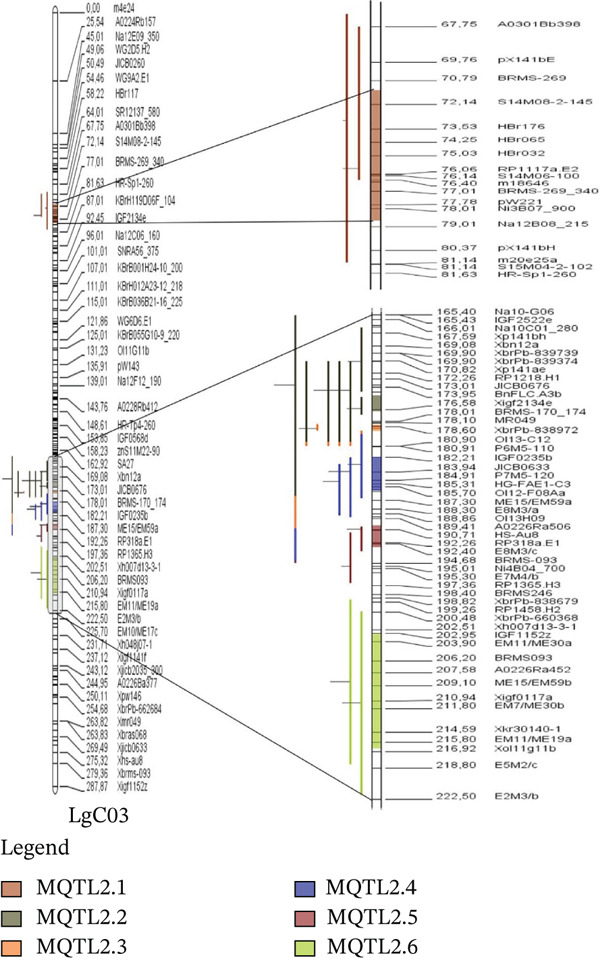
(b)

### 3.3. Identification of MQTLs

**Figure 3:**
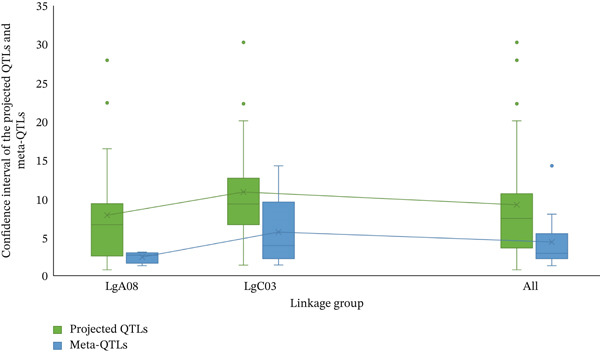
The graphical representation shows the projected QTLs and MQTLs on LgA08, LgC03, and across all combined linkage groups. The projected QTL confidence intervals are indicated in green, whereas the meta‐QTL confidence intervals are indicated in blue. Dots are used to represent outliers.

Veyrieras′ technique was used to identify the MQTL locations using the QTL projections and a freshly created consensus map. These MQTL regions are associated with desired traits where a minimum of two QTLs overlap. This overlap indicates that these regions are more likely to contain genes that influence the traits, providing a more refined and reliable understanding of the genomic regions involved. Consequently, 37 QTLs out of 57 were grouped into 10 MQTL areas. However, none of the 10 discovered MQTL areas could map 20 QTLs, which were either singlets or lacked overlapping regions. One MQTL region was rejected due to not having an overlapping section. On linkage group LgA08, four MQTLs were identified, and six MQTLs on linkage group LgC03 (Figure 2). The MQTLs′ CIs were considerably narrower than the CIs of the originally estimated QTLs, ranging from 0.8 to 13.59 cM in linkage group LgC03 and from 0.71 to 2.46 cM in linkage group LgA08 (Figure 3). The average CI of MQTLs for all linkage groups was 3.75 cM, a significant decrease from the predicted QTLs′ average CI of 8.56 cM. This reduction translates to a 2.28‐fold decrease, or a 56.20% reduction in the CI, making the MQTLs more precise. Each MQTL region is shown in Table 2 along with details on flanking markers, locations, 95% CIs, and the no. of QTLs associated with all MQTL. The number of QTLs has been reduced by 43.80% compared with the projected QTLs, resulting in a more concentrated grouping of QTLs that form highly stable MQTL regions with narrower CIs. The 10 identified MQTLs span a total distance of 44.17 cM across the two linkage groups, showing stability and reliability. These MQTLs, with their reduced CIs and increased stability, are promising candidates for further studies aimed at identifying specific genes associated with the traits of interest.

**Table 2:** An overview of the meta‐QTLs and total number of genes found in this investigation.

Meta‐QTLs	Linkage group	Position	Confidence interval (CI, 95%)	Flanking marker	No. of QTL in meta‐QTL	No. of genes
MQTL 1.1	LgA08	90.35	2.46	EM9/ME37a–EM2/ME3a	4	1
MQTL 1.2	LgA08	99.65	1.98	Niab090–Na12H07_300	9	10
MQTL 1.3	LgA08	103.17	2.14	JICB1088–BnGMS312	3	5
MQTL 1.4	LgA08	112.42	0.71	XbrPb‐840569–XbrPb‐660078	4	4
MQTL 2.1	LgC03	75.01	7.36	BRMS‐269–Na12B08_215	2	19
MQTL 2.2	LgC03	175.87	1.88	HBr014–BRMS‐170_174	7	12
MQTL 2.3	LgC03	178.81	0.8	BRAS068–OI13‐C12	1	2
MQTL 2.4	LgC03	184.2	4.01	JICB0633–OI12‐F08Aa	3	2
MQTL 2.5	LgC03	191.54	2.61	Na10‐C01–BRMS‐093	2	3
MQTL 2.6	LgC03	209.81	13.59	IGF1152z–E5M2/c	2	2

### 3.4. CGs Identification Within the MQTL Regions and Expression Analysis

Ten MQTL areas on two linkage groups (LgA08 and LgC03) had 67 genes in total, which were further studied. Each MQTL has between one (MQTL1.1) and 22 (MQTL2.2) genes identified. The region with the highest number of genes (21 genes) was the MQTL “MQTL2.2”, which was found on LgC03 at 175.87 cM (CI: 1.88 cM), followed by “MQTL2.1”, which had 18 genes. In contrast, just one gene was found in the MQTL “MQTL1.1,” which was located on LgA08 at 90.35 cM (CI: 2.46 cM). Out of the 67 genes, 17.91% (12) code for uncharacterized protein genes, proteins with uncertain functions, or domains with unknown functions (Table 3). Four and three genes, respectively, were found to be linked to the *FAE1*/Type III polyketide synthase‐like protein (3‐ketoacyl‐CoA synthase [KCS]), and the MYB domain protein (glucosinolate biosynthesis), across all MQTL areas. Additionally, two genes were linked to LUH, CPK9, gamma thionin domain, and TIFY8 domain proteins. Other domain proteins such as ZFP8, WD40, LUH, Polycomb protein, sucrose‐phosphate phosphatase (SPP), F‐box, and UBP8 were each associated with a single gene.

**Table 3:** High confidence candidate genes with their *Arabidopsis* sp. orthologs and function.

MQTLs	*B. napus* Genes	*Arabidopsis* orthologs	Description
MQTL 1.1	BnaA08g08440D	AT4G17785	MYB domain protein 39 (MYB39)
MQTL 1.2	BnaA08g14150D	AT4G27090	Ribosomal protein L14
	BnaA08g09950D	AT3G26010	Galactose oxidase/kelch repeat superfamily protein
	BnaA08g09940D	N/A	N/A
	BnaA08g11130D	AT4G34520	3ketoacylCoA synthase 18 (KCS18)
	BnaA08g27750D	AT1G04220	3ketoacylCoA synthase 2 (KCS2)
	BnaA08g11140D	AT4G34510	3ketoacylCoA synthase 17 (KCS17)
	BnaA08g12140D	AT4G32570	TIFY domain protein 8 (TIFY8)
	BnaA08g08160D	N/A	N/A
	BnaA08g08460D	AT4G17870	PYRABACTIN RESISTANCE 1 (PYR1)
	BnaA08g13570D	AT3G27835	Gamma thionin family protein
MQTL 1.3	BnaA08g07370D	AT5G32470	Haem oxygenase like, multihelical
	BnaA08g12140D	AT4G32570	TIFY domain protein 8 (TIFY8)
	A08p03080.1_BnaDAR	N/A	Sucrose phosphate like domain (SPP)
	BnaA08g13570D	AT3G27835	Gamma thionin family protein
	BnaA08g00020D	AT5G15660	F‐box and associated interaction domains containing protein
MQTL 1.4	BnaA08g13280D	AT4G29920	Double ClpN motif containing Ploop nucleoside triphosphate hydrolases superfamily protein
	BnaA08g08130D	AT4G16845	REDUCED VERNALIZATION RESPONSE 2 (VRN2)
	BnaA08g09210D	AT4G19500	Nucleoside tri phosphatases; transmembrane receptors; Nucleotide binding
	A08p04960.1_BnaDAR	N/A	ATP binding, Protein phosphorylation
MQTL 2.1	BnaC03g62830D	AT4G18770	MYB domain protein 98 (MYB98)
	BnaC03g17480D	AT2G31410	Unknown protein
	BnaC03g73670D	AT3G13040	MYB like HTH transcriptional regulator family protein
	BnaC03g16200D	AT5G50970	Transducing family protein/WD 40 repeat family protein
	BnaC03g16410D	AT5G50780	Histidine kinase, DNA gyrase B, and HSP90 like ATPase family protein
	BnaC03g17400D	AT2G31230	Ethylene responsive element binding factor 15 (ERF15)
	BnaC03g17360D‐	AT2G31150	ATP binding
	BnaC03g17320D	N/A	N/A
	BnaC03g17140D	AT2G30890	Cytochrome b561/ferric reductase transmembrane protein family
	BnaC03g70920D	AT1G55910	Zinc transporter 11 precursor (ZIP11);
	BnaC03g75930D	AT1G55860	E3 ubiquitin‐protein ligase UPL1 OS=*Arabidopsis* thaliana GN=UPL1 PE=1 SV=3encodes a ubiquitin‐protein ligase containing a HECT domain
	BnaC03g44030D	N/A	N/A
	BnaC03g10970D	AT5G22760	PHD finger family protein
	BnaC03g76920D	N/A	N/A
	BnaC03g06180D	AT5G14010	KNUCKLES (KNU)/ C2H2 zinc‐finger protein
	BnaC03g23550D	AT2G41940	Zinc finger protein 8 (ZFP8)
	BnaC03g07060D	AT5G01860	C2H2 and C2HC zinc fingers superfamily protein
	BnaC03g10610D	AT5G22030	Ubiquitin carboxyl‐terminal hydrolase 8
MQTL 2.2	BnaC03g41610D	AT3G20410	Calmodulin domain protein kinase 9 (CPK9)
	BnaC03g18050D	AT2G32700	LEUNIG_homolog (LUH)
	BnaC03g03590D	N/A	N/A
	BnaC03g08210D	AT1G25490	ROOTS CURL IN NPA (RCN1)
	BnaC03g08810D	AT5G18130	Unknown protein
	BnaC03g00140D	AT5G01620	TRICHOME BIREFRINGENCE LIKE 35
	BnaC03g00230D	AT3G45850	P loop containing nucleoside triphosphate hydrolases superfamily protein
	BnaC03g02370D	AT5G06110	DnaJ domain
	BnaC03g18050D	AT2G32700	LEUNIG_homolog (LUH)
	BnaC03g00050D	AT5G01760	ENTH/VHS/GAT family protein
	BnaC03g03480D	AT5G08330	TCP family transcription factor
	BnaC03g41310D	AT3G19970	Alpha/beta Hydrolases superfamily protein
	BnaC03g05120D	AT5G11890	Molecular_function unknown
	BnaC03g05820D	AT5G13330	Related to AP2 6l (Rap2.6L)
	BnaC03g06110D	AT5G13920	GRF zinc finger / Zinc knuckle protein
	BnaC03g09260D	N/A	N/A
	BnaC03g11110D	AT5G20480	EF TU receptor (EFR)
	BnaC03g12220D	AT3G44670	Disease resistance protein (TIR NBS LRR class) family
	BnaC03g18730D	AT2G33620	AT hook motif DNA binding family protein
	BnaC03g19820D	AT2G35940	BEL1 like homeodomain 1 (BLH1);
	BnaC03g67130D	AT4G32551	LEUNIG (LUG)
MQTL 2.3	BnaC03g70790D	AT4G02720	Unknown protein
	BnaC03g36370D	AT5G38680	Galactose oxidase/kelch repeat superfamily protein
MQTL 2.4	BnaC03g65980D	AT4G34520	Ketoacyl CoA synthase 18 (KCS18)
	BnaC03g64040D	AT4G21660	Proline rich spliceosome associated (PSP) family protein
MQTL 2.5	BnaC03g77520D	AT4G29760	Unknown protein
	BnaC03g77540D	AT4G29760	Unknown protein
	BnaC03g72770D	AT2G27170	ATP binding
MQTL 2.6	BnaC03g68570D	AT1G50380	Prolyl oligo peptidase family protein

**Figure 4:**
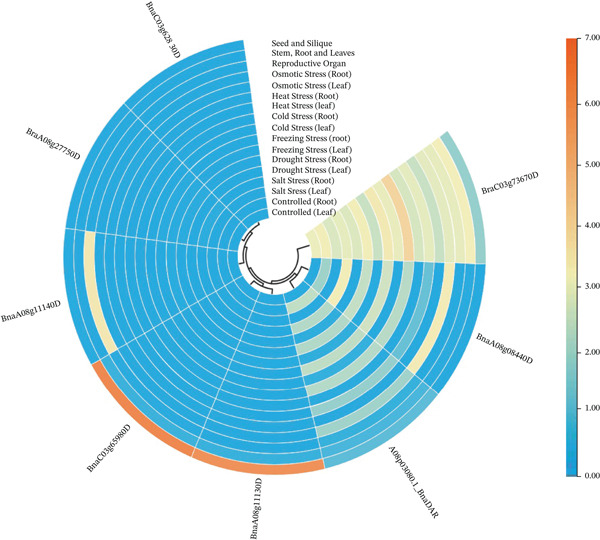
Heat map showing differential expression level of eight potential candidate genes underlying MQTL intervals.

**Figure 5:**
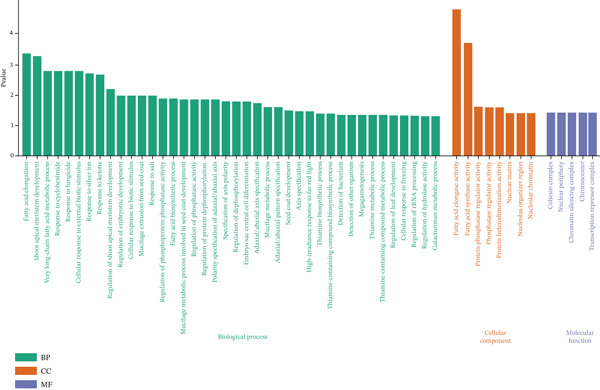
Gene ontology (GO) terms of the identified candidate genes.

**Figure 6:**
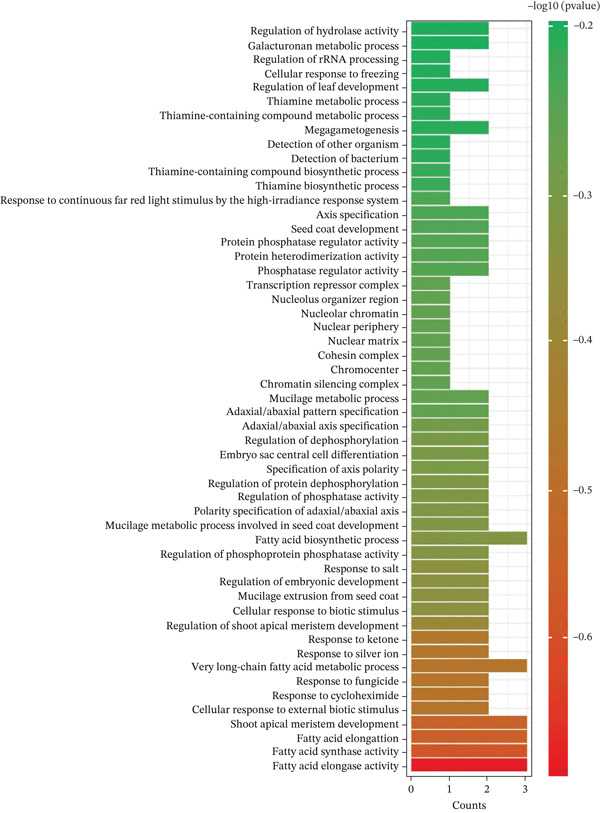
KEGG pathway enrichment of the identified candidate genes.

The expression patterns of the potential 8 genes were detected using the information resource of *B. napus* multiomics (https://yanglab.hzau.edu.cn/BnIR). As illustrated in Figure 4, the gene function data were divided into different parts like root, stem, leaf, reproductive organ, seed, and siliqua. These genes showed differential expression under different environmental conditions. Three categories were created from the GO analysis: BP, MF, and CC. Each category was designed to represent different facets of gene function and was visually represented accordingly (Figure 5). *p* values < 0.01 indicated that an expression was enriched in the corresponding gene set. The pathway annotations provided by KEGG enrichment analysis allowed for a thorough evaluation of the possible roles that these genes may play in various BPs. The analysis revealed that the most important pathway involved in the metabolic processes is synthetic activity related to fatty acids, compared with other pathways (Figure 6).

## 4. Discussion

Breeding efforts aimed at controlling or enhancing specific traits are crucial for the extensive cultivation and success of *B. napus* varieties. MQTL analysis is a robust strategy to refine consensus loci by integrating evidence across independent studies, thereby improving QTL localization and strengthening the reliability of marker‐trait associations (MTA) across diverse genetic backgrounds [39]. Although MQTL approaches have been applied in *B. napus* to refine loci for seed quality traits [40], root traits [41], yield‐related traits [42], and seed weight [43], an integrated synthesis of QTL evidence focused on EA content remains valuable for consolidating heterogeneous mapping results into stable consensus loci. Here, we integrate EA QTL information across studies to prioritize reproducible genomic regions and reduce confidence intervals for breeding application. Reducing EA content (< 2%) has long been a central breeding objective, yet the negative association between low‐EA alleles and seed oil content remains a practical constraint [44]. Lowered oil yield has been linked to low‐EA loci, potentially through pleiotropy or tight linkage [15, 45], whereas increased EA can coincide with higher oil content because elongation of oleic acid to VLCFAs increases the molecular mass of storage lipids [15, 46]. By refining the genomic positions of EA‐associated loci, our results provide a more precise foundation for marker development and for subsequent validation strategies aimed at improving oil quality while safeguarding oil yield.

This trade‐off is important for practical breeding because oil yield is a primary economic target that must be maintained while improving oil safety and quality. The reported decline in oil content in low‐EA backgrounds implies that selection for EA alone may inadvertently reduce total oil deposition, consistent with the pleiotropic effects reported for low‐EA loci [15, 45]. Conversely, the simultaneous increase of EA and total oil upon *FAE1* overexpression highlights tight metabolic coupling between VLCFA biosynthesis and overall oil accumulation [15, 46]. Therefore, developing LEAR with high‐oil content should be treated as a joint selection problem, and complementary loci that stabilize oil yield in low‐EA backgrounds are likely required [47]. In this context, the refined MQTL intervals and prioritized candidate genes reported here provide a more precise basis for marker development and downstream validation aimed at improving oil quality while safeguarding oil yields.

A comprehensive literature search identified 12 studies reporting 57 EA‐related QTLs, but only loci with adequate information for projection were retained. QTLs were excluded when projection showed poor model fit (high AIC) or when essential information was missing (insufficient common markers and/or absent flanking‐marker details). Ultimately, 37 QTLs were successfully projected (Figures 2a,b), generating 10 MQTLs with comparatively narrow CIs and thus tighter marker‐locus associations, consistent with Kumar et al. [48]. Across MQTLs, CIs were reduced 2.28‐fold (56.19%) relative to the projected QTLs, and the number of loci decreased by 43.80% after meta‐analysis (Figure 3; Table 2), resulting in more tightly delimited regions that are more actionable for marker development. Similar consolidation and CI reduction have been reported in MQTL studies of other major crops [35, 49–54].

Several features of the MQTLs support their utility for MAS. First, stability across environments was evident: MQTL1.2 was derived from nine EA QTLs across three studies [16, 24, 27] spanning more than four environments (Table 1), MQTL2.2 (LgC03), which integrates seven EA QTLs from different studies, further supports the presence of a recurrent genomic region influencing EA accumulation, and MQTL2.4 was derived from three EA QTLs across two studies [24, 31] conducted in multiple environments. Second, the detection of multiple MQTLs across LgA08 and LgC03 indicates that EA is governed by several reproducible genomic regions rather than a single major locus, providing multiple entry points for selection. Third, the potential for breeding trade‐offs is most relevant when EA‐associated MQTLs coincide with genes in pathways that are metabolically linked to overall oil deposition. In this study, MQTL1.2 (LgA08) and MQTL2.4 (LgC03) harbor KCS/*FAE1*‐pathway homologues (Table 3), which are well‐established as key components of VLCFA elongation and therefore directly implicated in EA biosynthesis [46]. Functional evidence further indicates that manipulation of *FAE1* can shift both EA profiles and total oil accumulation in some genetic backgrounds, supporting tight metabolic coupling between VLCFA elongation and seed oil traits [46]. Accordingly, these MQTL hotspots represent biologically plausible regions where selection for low‐EA alleles could have correlated effects on oil content through pleiotropy or tight linkage. Therefore, breeding LEAR with high‐oil content should be treated as a joint selection objective, and MQTL‐linked markers in such regions should be validated under multitrait selection to ensure that reduced EA is achieved without unintended penalties in oil yield [47].

Using EnsemblPlants genomic resources, we identified 67 CGs within the MQTL intervals (Table 3). These CGs were prioritized based on functional annotations relevant to EA biology and seed oil metabolism, with emphasis on (i) VLCFA elongation, (ii) pathways linked to seed quality traits such as glucosinolate‐related regulation, and (iii) sucrose metabolism as a major carbon source for fatty acid synthesis. Notably, four CGs encode 3‐KCS proteins implicated in fatty‐acid elongation and VLCFA metabolism: *BnaA08g11130D*, *BnaA08g11140D*, and *BnaA08g27750D* within MQTL1.2, and *BnaC03g65980D* within MQTL2.4 (Table 3; Figures 5 and 6). Among these, *BnaA08g11130D* and *BnaC03g65980D* showed relatively high expression in seed and silique tissues (Figure 4), supporting their candidacy for influencing EA accumulation. Because *FAE1* encodes a KCS enzyme and represents a key step in VLCFA elongation [46], KCS genes are established targets for modifying VLCFA profiles [55–63]. Consistent with this mechanism, LEAR/canola varieties show a near absence of VLCFAs, which has been linked to deficient KCS activity in microsomes [7]. Biochemically, synthesis of one EA molecule requires four acetyl‐CoA molecules [64]. Beyond elongation‐pathway candidates, we detected regulatory genes within certain MQTLs, including MYB‐domain transcription factors (e.g., MYB39: BnaA08g08440D in MQTL1.1; MYB98: BnaC03g62830D and BnaC03g73670D in MQTL2.1) (Table 3), which may influence seed metabolic programs and associated quality traits [65]. We also identified a sucrose‐metabolism candidate in MQTL1.3: A08p03080.1_BnaDAR, annotated with SPP activity. Because sucrose provides a major carbon input for fatty acid biosynthesis, this locus may indirectly influence EA accumulation by regulating sucrose‐derived carbon supply [66]. Overall, eight prioritized candidate genes associated directly or indirectly with EA‐related pathways were identified across MQTL1.1, MQTL1.2, MQTL1.3, MQTL2.1, and MQTL2.4 on LgA08 and LgC03 (Table 3). We also identified candidates annotated for carbohydrate metabolism and signaling, including kelch‐repeat proteins and sucrose‐pathway related genes [67–70], which currently represent secondary priorities relative to the KCS/*FAE1* pathway and warrant targeted validation.

Although MQTL analysis improves localization, interpretation should account for key sources of uncertainty. Publication bias may skew the input QTL set toward larger effects, and methodological heterogeneity among studies (population type, marker density, phenotyping environments, and QTL calling criteria) can affect comparability. Map projection onto a consensus framework also introduces residual positional uncertainty, and effect sizes from small populations may be inflated (Beavis effect) [71]. Accordingly, MQTL‐linked markers should be validated in independent, multienvironment datasets and updated as higher‐resolution linkage and GWAS resources accumulate.

Recent functional evidence supports the feasibility of directly targeting EA biosynthesis genes to achieve a canola‐quality threshold. Liu et al. [14] reported that suppressing *BnaFAE1* and *BnaFAD2* via RNA interference (RNAi) markedly reduced EA content in high‐erucic *B. napus* and further lowered EA in low‐erucic backgrounds, demonstrating strong pathway‐level control of EA accumulation. Similarly, intron‐spliced hairpin RNA (ihpRNA) targeting *FAE1* has been shown to substantially decrease EA levels, reinforcing the central role of the elongation pathway in determining EA profiles [63]. Consistent with this biological framework, our strongest MQTL hotspots (MQTL1.2 on LgA08 and MQTL2.4 on LgC03) harbor KCS/*FAE1*‐pathway candidates and define narrow, marker‐bounded intervals (Table 3; Figures 5 and 6), providing practical targets for MAS. Going forward, combining MQTL‐linked markers with functional validation of prioritized elongation‐pathway candidates, while simultaneously evaluating their effects under multitrait selection for EA and oil yield, will be essential for developing LEAR cultivars with improved oil quality while minimizing unintended trade‐offs in oil‐related traits.

## 5. Conclusion

In conclusion, we identified 10 MQTLs for EA content on two linkage groups (LgA08 and LgC03) and achieved substantially improved mapping precision compared with the projected QTLs. The mean CI decreased from 8.56 cM (projected QTLs) to 3.75 cM (MQTLs), representing a 56.19% reduction, and the number of loci was reduced by 43.80% following meta‐analysis. Collectively, these MQTLs span 44.17 cM across the two linkage groups and define narrower, marker‐bounded regions that facilitate candidate gene prioritization and downstream validation. Notably, MQTL1.2 and MQTL2.4 harbor KCS/FAE1‐pathway candidate genes, including 3‐KCS homologs that are central to very‐long‐chain fatty acid elongation and EA biosynthesis in *B. napus*. These refined MQTL intervals and associated markers provide a useful foundation for genomic‐assisted breeding and MAS aimed at developing LEAR cultivars with improved oil quality.

## Author Contributions

Md. Yeamin Hossain, Md. Ridwanul Islam, Mahfuj Ahmed, and Md. Harun‐Ur‐Rashid equally contributed to all the sections of this manuscript. Md. Harun‐Ur‐Rashid, Kazi Md. Kamrul Huda, and Shahanaz Parveen: supervised, reviewed, and edited the first draft of the manuscript. Mst. Sufara Akhter Banu and Masashi Inafuku: reviewed and edited the first draft of the manuscript.

## Funding

No funding was received for this manuscript.

## Disclosure

All authors approved the final manuscript.

## Ethics Statement

The research involved in the manuscript did not involve live vertebrates, higher invertebrates, or human subjects. Ethical considerations related to these elements are not applicable to this study.

## Conflicts of Interest

The authors declare no conflicts of interest.

## Supporting information


**Supporting Information** Additional supporting information can be found online in the Supporting Information section. Table S1: List of the markers analyzed under the study with their name and position. Table S2: Summary of the QTLs used for Meta‐QTLs study.

## Data Availability

All the data generated or analyzed during this study are included in the manuscript and supporting information files. Publicly available software was used in the study.
