# Supplementary material for: Unlocking the Genetic Blueprint of Erucic Acid Content in Mustard (Brassica napus): A Meta‐QTL Exploration
Source: ScientificWorldJournal. 2026 Mar 31;2026:8457904. doi: 10.1155/tswj/8457904 (PMC13140289; doi:10.1155/tswj/8457904)
Supplement: Supplementary file 1 — Supporting Information Additional supporting information can be found online in the Supporting Information section. Table S1: List of the markers analyzed under the study with their name and position. Table S2: Summary of the QTLs used for Meta‐QTLs study. [file TSWJ-2026-8457904-s001.docx]

**Supplementary Table 1** List of the markers analyzed under the study with their name and
position

| **Barret et al. 1995** | | |
| --- | --- | --- |
| **Chromosome** | **Locus Name** | **Position** |
| 9 | 1NF4b | 15.2 |
| 9 | 1ND1a | 11.9 |
| 9 | OPF01.390 | 0 |
| 9 | Lap-1 | 10 |
| 9 | OPG05.2090 | 19.9 |
| 9 | OPA15.850 | 32 |
| 9 | OPA08.960 | 22.1 |
| 9 | OPF14.770 | 40.7 |
| 9 | OPY04.1510 | 13.1 |
| 9 | OPG11.510 | 45.5 |
| 9 | Aco-1 | 26.1 |
| 9 | OPG13.720 | 10 |
| 9 | 5NA2a | 11.2 |
| 9 | OPY15.1270 | 11.9 |
| 9 | OPL08.790 | 15.9 |
| **Gandhi et al. 2003** | | |
| 2 | LS307 | 42.1 |
| 2 | LS427 | 56.7 |
| 2 | LS515 | 79.5 |
| 2 | LS196 | 23.7 |
| 2 | LS575 | 12.1 |
| 2 | LS454 | 67.5 |
| 2 | LS322 | 83.5 |
| 2 | LS424 | 0 |
| 2 | LS105 | 15.4 |
| 2 | LS90 | 72 |
| 2 | LS189 | 55.1 |
| 2 | LS265 | 75.7 |
| 3 | LS173 | 54.6 |
| 3 | LS20 | 42.5 |
| 3 | LS291B | 87.5 |
| 3 | LS108 | 0 |
| 3 | LS291A | 22.4 |
| 3 | LS136 | 50.8 |
| 3 | LS48 | 63.9 |
| 3 | LS333 | 53.5 |
| 3 | LS621 | 74.2 |
| 3 | LS187 | 77.4 |

**Supplementary Table 1 (continued)** List of the markers analyzed under the study with their name and
position

| **Gupta et al. 2004** | | |
| --- | --- | --- |
| **Chromosome** | **Locus Name** | **Position** |
| 3 | wg2a4a | 0 |
| 3 | e39m55h163 | 7.1 |
| 3 | p65t76v198 | 20.2 |
| 3 | p79t78v127 | 29.2 |
| 3 | p65m62h265 | 33.3 |
| 3 | e54t75h129 | 40.9 |
| 3 | e47t64v352 | 45.9 |
| 3 | e39m50h446 | 54.8 |
| 3 | p62t78h185 | 63.11 |
| 3 | p53t76h368 | 68.4 |
| 3 | FAE1.2 | 72.6 |
| 3 | e79t78v205 | 76.1 |
| 3 | e50t67v375 | 87.1 |
| 3 | p32t77v268 | 110.1 |
| 3 | p65m62h181 | 118.4 |
| 3 | e31m50v273 | 122.9 |
| 17 | e31m60v98 | 0 |
| 17 | e48t78v400 | 3.6 |
| 17 | e34m50v82 | 12.6 |
| 17 | wg2h1 | 19.4 |
| 17 | p62m39v124 | 27.8 |
| 17 | e39m50v500 | 36 |
| 17 | e54t71h380 | 46.7 |
| 17 | e32166h142 | 49.2 |
| 17 | e32166v191FA | 50.1 |
| 17 | e31m60v195 | 52.6 |
| 17 | p65t90v258 | 59.9 |
| 17 | e39m50v500 | 36 |
| 17 | e54t71h380 | 46.7 |
| 17 | e32166h142 | 49.2 |
| 17 | e32166v191FA | 50.1 |
| 17 | e31m60v195 | 52.6 |
| 17 | p65t90v258 | 59.9 |

**Supplementary Table 1 (continued)** List of the markers analyzed under the study with their name and
position

| **Chromosome** | **Locus Name** | **Position** |
| --- | --- | --- |
| **Qiud et al.2006** | | |
| 1 | S15M04-2-330 | 45 |
| 1 | pW1990a | 95 |
| 1 | sN11641 | 17 |
| 1 | SA63 | 72 |
| 1 | IGF3222b | 18 |
| 1 | P4M4-370 | 77 |
| 1 | RA2G09 | 53 |
| 1 | IGF0557c | 67 |
| 1 | S12M03-2-85 | 32 |
| 1 | pW145 | 44 |
| 1 | E5HM31-505 | 61 |
| 1 | E10HM34-230 | 85 |
| 1 | P8M8-220 | 0 |
| 1 | pW248 | 29 |
| 1 | IGF9014b | 65 |
| 1 | E8HM40-150 | 14 |
| 1 | pW157 | 27 |
| 1 | S13M08-1-70 | 82 |
| 1 | RA2E04 | 70 |
| 1 | IGF2071e | 74 |
| 2 | 0113E08 | 30 |
| 2 | S14M06-150 | 79 |
| 2 | IGF5224g | 22 |
| 2 | S10M16-2-210 | 49 |
| 2 | P8M3-295 | 116 |
| 2 | IGF1086e | 76 |
| 2 | S08M15-170 | 54 |
| 2 | S05M05-250 | 64 |
| 2 | E1HM33-300 | 84 |
| 2 | P4M4-120 | 142 |
| 2 | E5HM40-310 | 0 |
| 2 | P10M8-300 | 36 |
| 2 | pX155 | 55 |
| 2 | 0110F04 | 12 |
| 2 | S04M03-1-180 | 69 |
| 2 | S10M03-1-280 | 46 |
| 8 | sR7178 | 18 |
| 8 | pW184 | 89 |
| 8 | P7M5-170 | 0 |
| 8 | RA2E12 | 23 |
| 8 | Na12B05a | 43 |
| 8 | IGF5276b | 71 |
| 8 | sS1702 | 21 |
| 8 | IGF1108c | 35 |
| 13 | S16M10-217 | 0 |
| 13 | S15M04-2-102 | 19 |
| 13 | P6M5-110 | 116 |
| 13 | IGF2134e | 32 |
| 13 | P7M5-120 | 120 |
| 13 | IGF0117a | 64 |
| 13 | IGF1141f | 87 |
| 13 | STS02 | 28 |
| 13 | E10HM32-140 | 106 |
| 13 | BN12A | 23 |
| 13 | IGF3165b | 80 |
| 13 | E7HM31-450 | 90 |
| 13 | IGF 2522e | 98 |
| 13 | S04M03-1-170 | 49 |
| 13 | IGF5376b | 102 |
| 13 | S14M08-2-145 | 10 |
| 13 | S14M06-100 | 14 |

**Supplementary Table 1 (continued)** List of the markers analyzed under the study with their name and
position

| **Chromosome** | **Locus Name** | **Position** |
| --- | --- | --- |
| **Amar et al. 2008** | | |
| 8 | OPA15.896 | 35.7 |
| 8 | OPS7.970 | 66.7 |
| 8 | RP1457.H2 | 20 |
| 8 | RP1602.H1 | 21.2 |
| 8 | RP1227.E1 | 0 |
| 8 | GATA.H3 | 62.9 |
| 8 | OPD3.1055 | 70 |
| 8 | OPQ9.1590 | 77.9 |
| 8 | MG20 | 6.1 |
| 8 | MG21 | 46.5 |
| 8 | MG23 | 67.9 |
| 13 | OPAG10.630 | 129.5 |
| 13 | WG1G8.H1 | 94.5 |
| 13 | RP1471.H1 | 33.9 |
| 13 | RP1218.H1 | 123.2 |
| 13 | WG5B1.H1 | 51.1 |
| 13 | RP459.H1 | 106.8 |
| 13 | RP1365.H3 | 148.3 |
| 13 | RP1477.E1 | 102.6 |
| 13 | WG2D5.H2 | 0 |
| 13 | RP1458.H2 | 150.2 |
| 13 | WG9A2.E1 | 5.4 |
| 13 | RP318a.E1 | 143.2 |
| 13 | RP1117a.E2 | 27 |
| 13 | WG6D6.E1 | 72.8 |

**Supplementary Table 1 (continued)** List of the markers analyzed under the study with their name and
position

| **Chromosome** | **Locus Name** | **Position** |
| --- | --- | --- |
| **Zhang et al. 2008** | | |
| 8 | m14e24a | 0 |
| 8 | m11e37b | 16.82 |
| 8 | A02268a267 | 31.77 |
| 8 | Ra1-F06 | 68.53 |
| 8 | Ra2-004 | 71.98 |
| 8 | A0302Rb405 | 91.93 |
| 8 | A0215Gb191 | 126.93 |
| 8 | A0216Gb206 | 144.02 |
| 8 | m5e42 | 171.83 |
| 8 | A0226Gb468 | 193.96 |
| 8 | m29e20 | 208.38 |
| 8 | m19e20 | 212.46 |
| 13 | A0215Rb317 | 0 |
| 13 | m4e24 | 13.72 |
| 13 | A0224Rb157 | 39.26 |
| 13 | A0301QGb399 | 73.24 |
| 13 | A0301Bb398 | 81.47 |
| 13 | m18646 | 90.12 |
| 13 | m20e25a | 94.86 |
| 13 | A0216Gb190 | 107.81 |
| 13 | A0214Ra310 | 115.68 |
| 13 | m24e45 | 129.3 |
| 13 | m20e46b | 132.83 |
| 13 | A0228Rb412 | 157.48 |
| 13 | Na12-G05a | 164.64 |
| 13 | Na12-G05b | 168.13 |
| 13 | A0226Ra506 | 203.13 |
| 13 | A0226Ra452 | 221.3 |
| 13 | A0226Ga438 | 242.41 |
| 13 | A0226Ba438 | 252.14 |
| 13 | A0226Ba377 | 258.67 |
| 13 | A0226Ba367 | 268.4 |
| 13 | A0226Ra425 | 286.48 |
| **Cao et al. 2010** | | |
| 8 | HBr026 | 3.32 |
| 8 | HR-S4-295 | 15.9 |
| 8 | CNU090 | 17.26 |
| 8 | sR7178 | 19.41 |
| 8 | HBr015 | 20.71 |
| 8 | sS1702 | 21.85 |
| 8 | FAE1-AS | 22.79 |
| 8 | JICB1088 | 23.42 |
| 8 | Ra2E12 | 24.04 |

**Supplementary Table 1 (continued)** List of the markers analyzed under the study with their name and
position

| **Chromosome** | **Locus Name** | **Position** |
| --- | --- | --- |
| **Smooker et al. 2010** | | |
| 8 | Ni4F06_200 | 0 |
| 8 | BRMS-006_195 | 1 |
| 8 | Ra1F06_180 | 123 |
| 8 | Na12H09_600 | 36 |
| 8 | sR3688_370 | 37 |
| 8 | Na12B11_600 | 38 |
| 8 | BRMS-319_338 | 39 |
| 8 | BRMS-176_243 | 39 |
| 8 | SN12352_340 | 42 |
| 8 | BRMS-088_250 | 52 |
| 8 | Na12H07_300 | 54 |
| 8 | JICB0633 | 55 |
| 8 | sS1702_370 | 57 |
| 8 | SR7178_310 | 60 |
| 8 | BRMS-094_290 | 61 |
| 8 | BRMS-013_350 | 62 |
| 8 | Na10C08_110 | 64 |
| 13 | Na12E09_350 | 0 |
| 13 | BRMS-229_295 | 7 |
| 13 | BRMS-006_160 | 12 |
| 13 | BRMS-221_295 | 14 |
| 13 | BRMS-218_295 | 14 |
| 13 | SR12137_580 | 19 |
| 13 | BRMS-071 | 32 |
| 13 | BRMS-269_340 | 32 |
| 13 | Ni3B07_900 | 33 |
| 13 | Na12B08_215 | 34 |
| 13 | sR6688_450 | 40 |
| 13 | KBrH119D06F_104 | 42 |
| 13 | JICB0634 | 43 |
| 13 | OI10B06_285 | 50 |
| 13 | Ni4F06_050 | 50 |
| 13 | Na12C06_160 | 51 |
| 13 | SNRA56_375 | 56 |
| 13 | KBrB001H24-10_200 | 62 |
| 13 | KBrB001H24-18_224 | 62 |
| 13 | Na10D03_180 | 65 |
| 13 | KBrB043L02-12_204 | 65 |
| 13 | KBrH012A23-12_218 | 66 |
| 13 | KBrB036B21-16_225 | 70 |
| 13 | BRMS-287_254 | 71 |
| 13 | Ra2H08_195 p | 74 |
| 13 | KBrB055G10-9_220 | 80 |
| 13 | Na12F12_190 | 94 |
| 13 | Na10C08_130 | 101 |
| 13 | Na12A08_280 | 107 |
| 13 | Ni4D10_075 | 107 |
| 13 | sN2032_420 | 107 |
| 13 | Ni2C03_300 | 108 |
| 13 | Na12A07_185 | 110 |
| 13 | Na10C01_280 | 121 |
| 13 | JICB0676 | 128 |
| 13 | BRMS-170_174 | 133 |
| 13 | BRMS-214 250 | 141 |
| 13 | Ni4B04_700 | 150 |

**Supplementary Table 1 (continued)** List of the markers analyzed under the study with their name and
position

| **Chromosome** | **Locus Name** | **Position** |
| --- | --- | --- |
| **Yan et al. 2010** | | |
| 8 | EM1/ME16 | 0 |
| 8 | EM1/ME8a | 1.9 |
| 8 | EM10/ME17a | 5.6 |
| 8 | E4M5/b | 17.6 |
| 8 | EM8/ME34a | 11.2 |
| 8 | E4M6/b | 114.2 |
| 8 | E4M5/c | 22.1 |
| 8 | CB10530a | 24.3 |
| 8 | ME16/EM10 | 27.2 |
| 8 | ME16/EM50c | 28.3 |
| 8 | EM10/ME36a | 29.2 |
| 8 | FAE1/ME42 | 29.5 |
| 8 | E7M4/e | 131.9 |
| 8 | ME16/EM50b | 33.1 |
| 8 | SR9411 | 136.1 |
| 8 | E4M6/a | 138 |
| 8 | EM8/ME14 | 39 |
| 8 | 0112-F08Ab | 41.2 |
| 8 | RuBPc/EM64d | 42.8 |
| 8 | CB10364 | 45.2 |
| 8 | EM9/ME37a | 47.5 |
| 8 | EM6/ME30d | 48.4 |
| 8 | EM2/ME3c | 49 |
| 8 | sS2331Ba | 49.8 |
| 8 | CB10578 | 52.7 |
| 8 | SS1949 | 156.8 |
| 8 | BRMS013 | 60.8 |
| 8 | ME16/EM12c | 62.4 |
| 8 | BRMS342 | 66.8 |
| 13 | Na12-G05 | 0 |
| 13 | E7M4/c | 8.2 |
| 13 | Na10-E02b | 11.2 |
| 13 | EM11/ME30e | 13.4 |
| 13 | ME16/EM64 | 15.9 |
| 13 | EM10/ME13a | 19.1 |
| 13 | Na10-G06 | 21.9 |
| 13 | EM10/ME28 | 30.2 |
| 13 | MR049 | 34.6 |
| 13 | OI13-C12 | 37.4 |
| 13 | OI12-F08Aa | 42.2 |
| 13 | ME15/EM59a | 43.8 |
| 13 | E8M3/a | 44.8 |
| 13 | ME16/EM32d | 45.7 |
| 13 | Na10-C01 | 47.3 |
| 13 | E8M3/c | 48.9 |
| 13 | E7M4/b | 51.8 |
| 13 | BRMS246 | 54.9 |
| 13 | EM11/ME30a | 60.4 |
| 13 | BRMS093 | 62.7 |
| 13 | ME15/EM59b | 65.6 |
| 13 | EM7/ME30b | 68.3 |
| 13 | EM11/ME19a | 72.3 |
| 13 | E5M2/c | 75.3 |
| 13 | E2M3/b | 79 |
| 13 | EM10/ME17c | 82.2 |

**Supplementary Table 1 (continued)** List of the markers analyzed under the study with their name and
position

| **Chromosome** | **Locus Name** | **Position** |
| --- | --- | --- |
| **Wang et al. 2013** | | |
| 8 | elm19-100 | 0 |
| 8 | e8m13-117 | 11.4 |
| 8 | S008P19-1 | 15.7 |
| 8 | FITO131 | 17.8 |
| 8 | e6m 28-337 | 20.3 |
| 8 | sS1702 | 21.4 |
| 8 | BnGMS389 | 25.9 |
| 8 | H004105-1 | 28.9 |
| 8 | BnGMS312 | 30.5 |
| 8 | CB10629 | 42.9 |
| 6 | BRMS-027a | 0 |
| 6 | CB10143 | 1.1 |
| 6 | e21m6-110 | 13 |
| 6 | Nal 2D08 | 22.7 |
| 6 | O110D01 | 28.6 |
| 6 | e6m 18-400 | 29.7 |
| 6 | BRAS052a | 30.5 |
| 6 | B086M23 | 34.6 |
| 6 | niab134 | 35.5 |
| 6 | Ra2D04 | 36.9 |
| 6 | RA1F06 | 36.9 |
| 6 | BRMS-30 | 37.7 |
| 6 | e8m23-342 | 48.9 |
| 6 | BnGMS480 | 49.9 |
| 6 | CB10006 | 50.9 |
| 6 | Nal2C01b | 52.2 |
| 6 | BnGMS650 | 52.3 |
| 6 | CB10065 | 52.5 |
| 6 | Nal 2A08b | 52.8 |
| 6 | BnGMS317 | 53.1 |
| 6 | Nal2A08 a | 53.2 |
| 6 | el7m15-139 | 54.2 |
| 6 | Nal2B08 | 60.4 |
| 6 | Nal2H07b | 62.4 |
| 6 | CB10121 | 64.3 |
| 6 | S013H10-1 | 69.3 |
| 6 | sN12508 | 71.1 |
| 6 | BRAS014a | 77.2 |
| 6 | el7m15-141 | 78.3 |
| 6 | e6m 25-125 | 85.6 |
| 6 | niab041 | 91.7 |
| 6 | CB10101 | 93.4 |
| 6 | HAU77-3 | 95.1 |
| 6 | CB10330 | 109.5 |
| 6 | Ra3C04 | 112.1 |
| 6 | B057L05-1 | 117.8 |

**Supplementary Table 1 (continued)** List of the markers analyzed under the study with their name and
position

| **Chromosome** | **Locus Name** | **Position** |
| --- | --- | --- |
| **Jabidfar and Cheng 2013** | | |
| 3 | At1g03180 | 0 |
| 3 | BnapPIP1353 | 0.8 |
| 3 | At1g16740 | 3.4 |
| 3 | SB2121A | 5 |
| 3 | At1g09760 | 7.9 |
| 3 | Atlg10840 | 9.7 |
| 3 | Atlg18340a | 20.9 |
| 3 | BnapPIP86 | 21.1 |
| 3 | BnapPIP1367 | 21.2 |
| 3 | BnapPIP11b | 22.1 |
| 3 | BrapPIP184 | 27.5 |
| 3 | BnapPIP922 | 28.1 |
| 3 | BnapPIP873a | 29.9 |
| 3 | sB2771 | 36.4 |
| 3 | BnapPIP297 | 43.1 |
| 3 | At4g28200 | 46.5 |
| 3 | BnapPIP890 | 49.7 |
| 3 | At4g34700c | 56.8 |
| 3 | BnapPIP948 | 60.7 |
| 3 | BnapPIP1621 | 67.7 |
| 3 | BnapPIP1093 | 69 |
| 3 | At2g12462a | 69.7 |
| 3 | BnapPIP489a | 70.5 |
| 3 | BnapPIP1259 | 71.2 |
| 3 | At1g34350 | 71.2 |
| 3 | BrapPIP36 | 71.6 |
| 3 | BnapPIP835 | 71.7 |
| 3 | BnapPIP999 | 75.7 |
| 3 | BnapPIP143 | 81.7 |

**Supplementary Table 1 (continued)** List of the markers analyzed under the study with their name and
position

| **Chromosome** | **Locus Name** | **Position** |
| --- | --- | --- |
| **Wang et al. 2015** | | |
| 7 | znS13M26-340 | 58.97 |
| 7 | CNU331 | 89.24 |
| 7 | CNU053b | 103.48 |
| 7 | HBr021 | 78.32 |
| 7 | CNU339 | 80.41 |
| 7 | JICB0553 | 94.75 |
| 7 | niab043 | 61.37 |
| 7 | HR-C011-A7b | 21.57 |
| 7 | T23 | 75.74 |
| 7 | IGF0504f | 66.56 |
| 7 | BOM65 | 70.71 |
| 7 | IGF2036b | 18.59 |
| 7 | CNU063 | 16.95 |
| 7 | BRAS023 | 27.85 |
| 7 | znS06M34-50 | 70 |
| 7 | HBr009 | 99.59 |
| 7 | IGF1226l | 100.36 |
| 7 | JICKBrB027O09-8 | 54.71 |
| 7 | IGF1226f | 100.8 |
| 7 | JICB0011 | 100.2 |
| 7 | JICB0571 | 67.72 |
| 7 | BRMS-129 | 20.96 |
| 7 | IGF1226a | 110 |
| 7 | RPSaA | 69.05 |
| 8 | BRMS-088 | 70.12 |
| 8 | HBr120 | 30.07 |
| 8 | JICB0335 | 23.67 |
| 8 | HR-S4-295 | 78.25 |
| 8 | HBr022 | 68.04 |
| 8 | HBr106 | 26.76 |
| 8 | HBr105 | 30.37 |
| 8 | HBr104 | 36.76 |
| 8 | sS1702 | 71.69 |
| 8 | JICB0018 | 85.41 |
| 8 | HBr026 | 89.04 |
| 8 | HG4-SC9TN | 12.56 |
| 8 | HR-C002-A8 | 80.29 |
| 8 | IGF1108c | 57.53 |
| 8 | sR7178 | 74.24 |
| 8 | BRMS-176 | 44.82 |
| 8 | HBr010 | 41.16 |
| 8 | HBr031 | 51.28 |
| 8 | HBr074 | 73.74 |
| 8 | HBr095 | 73.13 |
| 8 | HR-Tp3-390 | 94.3 |
| 8 | em10me26-120 | 19.25 |
| 8 | CNU489 | 93.09 |
| 8 | HBr017 | 31.57 |
| 8 | HBr016 | 74.99 |
| 8 | HBr015 | 75.51 |
| 8 | HBr107 | 28.42 |
| 8 | niab090 | 66.75 |
| 8 | HG-FAE1-A8 | 70.68 |
| 8 | CNU208 | 55.34 |
| 8 | Na12B05a | 47.95 |
| 8 | RA2E12 | 69.42 |
| 8 | CNU090 | 77.41 |
| 8 | IGF5276b | 0 |
| 8 | HG4-SCD2 | 6.64 |
| 13 | CNU099 | 97.95 |
| 13 | pX141bH | 29.88 |
| 13 | HR-Sp1-260 | 31.14 |
| 13 | JICB2035_300 | 110.77 |
| 13 | HBr065 | 23.76 |
| 13 | pX141bE | 19.27 |
| 13 | HBr062 | 95.98 |
| 13 | em09me10-100 | 44.28 |
| 13 | JICB2001_300 | 114.5 |
| 13 | JICB0633 | 135.52 |
| 13 | HR-S2-360 | 30.67 |
| 13 | MR049 | 129.68 |
| 13 | IGF3165b | 92.86 |
| 13 | HG-FLC-C3b | 38.64 |
| 13 | IGF0568d | 104.98 |
| 13 | IGF5376b | 118.49 |
| 13 | Na10G06b | 115.57 |
| 13 | pX141aE | 32.93 |
| 13 | HBr052 | 65.88 |
| 13 | HS-Au8 | 142.29 |
| 13 | em12me29-280 | 36.81 |
| 13 | HBr051 | 67.62 |
| 13 | HBr014 | 126.1 |
| 13 | HBr211 | 52.66 |
| 13 | IGF0235b | 133.79 |
| 13 | HBr056 | 50.46 |
| 13 | HBr176 | 23.04 |
| 13 | JICB2040_200 | 91.66 |
| 13 | BRMS-093 | 146.26 |
| 13 | HBr139 | 94.07 |

**Supplementary Table 1 (continued)** List of the markers analyzed under the study with their name and
position

| **Chromosome** | **Locus Name** | **Position** |
| --- | --- | --- |
| 13 | JICB2047_250 | 115.85 |
| 13 | HR-Tp4-260 | 99.26 |
| 13 | Ol13H09 | 140.44 |
| 13 | BN12A | 34.65 |
| 13 | BRAS068 | 129.74 |
| 13 | em21me20-360 | 62.95 |
| 13 | SA27 | 114.38 |
| 13 | IGF1152z | 154.53 |
| 13 | HBr083 | 78.59 |
| 13 | HBr085 | 8.04 |
| 13 | BRMS-269 | 20.3 |
| 13 | IGF2134e | 41.01 |
| 13 | IGF2522e | 116.97 |
| 13 | HBr161 | 63.79 |
| 13 | sN2032 | 114.97 |
| 13 | Ol13C12 | 130.36 |
| 13 | IGF0117a | 74.77 |
| 13 | IGF1141f | 102.3 |
| 13 | JICB0260 | 0 |
| 13 | JICB0220 | 47.38 |
| 13 | BRMS-218 | 5.95 |
| 13 | em15me02-280 | 104.22 |
| 13 | HBr032 | 24.54 |
| 13 | HBr152 | 58.95 |
| 13 | BRMS-071 | 26.52 |
| 13 | HBr117 | 7.73 |
| 13 | HG-FAE1-C3 | 136.89 |
| 13 | znS11M22-90 | 109.53 |
| 13 | Ol11G11b | 80.59 |
| 13 | BRMS-106 | 26 |
| 13 | pW221 | 27.29 |
| 13 | pW143 | 85.47 |
| 13 | pW146 | 118.27 |

**Supplementary Table 2** Summary of the QTLs used for Meta-QTLs study

| **QTLs** | **Linkage group** | **C.I. (from)** | **C.I. (to)** | **LOD** | **R^2^** | **Reference** |
| --- | --- | --- | --- | --- | --- | --- |
| Barret_1998_qEA1 | LgA09 | 15.9 | 19.9 | 3.01 | 0.104 | Barret et al. 1998 |
| Cao_2010_qEA1 | LgA08 | 22.67 | 22.93 | 3.5 | 0.387 | Cao et al. 2010 |
| Cao_2010_qEA2 | LgA08 | 15.82 | 15.98 | 3.5 | 0.324 |  |
| Ecke_2008_qEA1 | LgA08 | 56.0 | 64.0 | 1.5 | 0.47 | Amar et al. 2008 |
| Ecke_2008_qEA2 | LgA08 | 54.0 | 60.0 | 3.0 | 0.39 |  |
| Ecke_2008_qEA3 | LgA08 | 58.0 | 64.0 | 2.6 | 0.31 |  |
| Ecke_2008_qEA4 | LgA08 | 60.0 | 66.0 | 1.85 | 0.13 |  |
| Ecke_2008_qEA5 | LgA08 | 34.0 | 40.0 | 1.2 | 0.2 |  |
| Ecke_2008_qEA6 | LgC03 | 134.0 | 140.0 | 2.7 | 0.34 |  |
| Ecke_2008_qEA7 | LgC03 | 130.0 | 138.0 | 2.9 | 0.29 |  |
| Ecke_2008_qEA8 | LgC03 | 32.0 | 18.0 | 2.1 | 0.06 |  |
| Ecke_2008_qEA9 | LgC03 | 142.0 | 148.0 | 1.92 | 0.19 |  |
| Farzad_2013_qEA | LgA03 | 49.7 | 56.8 | 3.8 | 0.923 | Javidfar and Cheng 2013 |
| Gandhi_2003_qEA1 | LgA02 | 0.0 | 12.1 | 1.57 | 0.01224 | Gandhi et al. 2003 |
| Gandhi_2003_qEA2 | LgA02 | 12.1 | 15.4 | 1.64 | 0.217 |  |
| Gandhi_2003_qEA3 | LgA02 | 0 | 12.1 | 0.95 | 0.01154 |  |
| Gandhi_2003_qEA4 | LgA03 | 54.6 | 63.9 | 1.34 | 0.131 |  |
| Gandhi_2003_qEA5 | LgA03 | 77.4 | 87.5 | 1.38 | 0.149 |  |
| Gupta_2004_qEA1 | LgA03 | 68.4 | 76.1 | 4.6 | 0.60 | Gupta et al. 2004 |
| Gupta_2004_qEA2 | LgA17 | 49.2 | 52.6 | 6.1 | 0.38 |  |
| QiuD_2006_qEA1 | LgA01 | 70.0 | 82.0 | 1.0 | 0.061 | Qiu et al. 2006 |
| QiuD_2006_qEA2 | LgA01 | 70.0 | 72.0 | 1.0 | 0.096 |  |
| QiuD_2006_qEA3 | LgA01 | 70.0 | 72.0 | 1.0 | 0.119 |  |
| QiuD_2006_qEA4 | LgA01 | 77 | 82.0 | 1.0 | 0.111 |  |
| QiuD_2006_qEA5 | LgA02 | 84 | 116 | 1.0 | 0.049 |  |
| QiuD_2006_qEA6 | LgA02 | 84 | 116 | 1.0 | 0.065 |  |
| QiuD_2006_qEA7 | LgA02 | 84 | 116 | 1.0 | 0.045 |  |
| QiuD_2006_qEA8 | LgA02 | 84 | 116 | 1.0 | 0.063 |  |
| QiuD_2006_qEA9 | LgA08 | 18.0 | 21.0 | 5.46 | 0.391 |  |
| QiuD_2006_qEA10 | LgA08 | 18.0 | 21.0 | 6.27 | 0.444 |  |
| QiuD_2006_qEA11 | LgA08 | 18.0 | 21.0 | 6.29 | 0.441 |  |
| QiuD_2006_qEA12 | LgA08 | 18.0 | 23.0 | 6.38 | 0.471 |  |
| QiuD_2006_qEA13 | LgC03 | 106.0 | 116.0 | 4.8 | 0.327 |  |
| QiuD_2006_qEA14 | LgC03 | 106.0 | 116.0 | 4.94 | NA |  |
| QiuD_2006_qEA15 | LgC03 | 106.0 | 116.0 | 4.83 | 0.283 |  |
| QiuD_2006_qEA16 | LgC03 | 106.0 | 116.0 | 4.8 | 0.304 |  |
| Smooker_2010_qEA1 | LgA08 | 37.3 | 64.5 | 0.0 | 0.378 | Smooker et al. 2010 |
| Smooker_2010_qEA2 | LgC03 | 120.5 | 150.0 | 0.0 | 0.458 |  |
| Wang_2013_qEA1 | LgA08 | 28.9 | 30.5 | 2.5 | 0.046 | Wang et al. 2013 |
| Wang_2013_qEA2 | LgA08 | 15.7 | 17.8 | 2.5 | 0.067 |  |

**Supplementary Table 2 (Continued).** Summary of the QTLs used for Meta-QTLs study

| **QTLs** | **Linkage group** | **C.I. (from)** | **C.I. (to)** | **LOD** | **R^2^** | **Reference** |
| --- | --- | --- | --- | --- | --- | --- |
| Wang_2015_qEA1 | LgA07 | 100.2 | 105.5 | 0.966 | 0.0127 | Wang et al. 2015 |
| Wang_2015_qEA2 | LgA08 | 58.6 | 68.0 | 2.358 | 0.19 |  |
| Wang_2015_qEA3 | LgA08 | 69.9 | 71.7 | 3.55 | 0.397 |  |
| Wang_2015_qEA4 | LgA08 | 73.1 | 74.6 | 2.38 | 0.17 |  |
| Wang_2015_qEA5 | LgA08 | 76.6 | 78.3 | 2.47 | 0.3 |  |
| Wang_2015_qEA6 | LgA08 | 80.3 | 86.8 | 3.04 | 0.31 |  |
| Wang_2015_qEA7 | LgC03 | 118.5 | 126.1 | 2.27 | 0.29 |  |
| Wang_2015_qEA8 | LgC03 | 126.7 | 128.8 | 2.34 | 0.3 |  |
| Wang_2015_qEA9 | LgC03 | 129.9 | 130.7 | 3.17 | 0.38 |  |
| Wang_2015_qEA10 | LgC03 | 133.8 | 140.9 | 3.62 | 0.46 |  |
| Wang_2015_qEA11 | LgC03 | 142.0 | 144.9 | 2.84 | 0.3 |  |
| Yan_2010_qEA1 | LgA08 | 25.7 | 47.4 | 2.1 | 0.305 | Yan et al. 2010 |
| Yan_2010_qEA2 | LgA08 | 29.5 | 45.3 | 2.1 | 0.391 |  |
| Yan_2010_qEA3 | LgC03 | 56.9 | 78.5 | 2.1 | 0.416 |  |
| Yan_2010_qEA4 | LgC03 | 55.5 | 74.9 | 2.1 | 0.437 |  |
| Zhang_2008_qEA1 | LgA08 | 16.82 | 31.77 | 2.7 | 0.16 | Zhang et al. 2008 |
| Zhang_2008_qEA2 | LgC03 | 81.47 | 90.12 | 3.2 | 0.31 |  |
